# Docking and ADMET studies for investigating the anticancer potency of Moscatilin on APC10/DOC1 and PKM2 against five clinical drugs

**DOI:** 10.1186/s43141-021-00256-6

**Published:** 2021-10-19

**Authors:** Ipsita Pujari, Ritobrata Sengupta, Vidhu Sankar Babu

**Affiliations:** Department of Plant Sciences, Manipal School of Life Sciences, Manipal Academy of Higher Education, Manipal, Udupi, Karnataka 576104 India

**Keywords:** ADMET, APC10/DOC1, Docking, Moscatilin, PKM2

## Abstract

**Background:**

Moscatilin is a bibenzyl derivative (stilbenoid), mainly found in *Dendrobium* species. This plant-derived chemical is a potential cytotoxic anticancer drug that acts against different cancer types. The present study compared the structural interactions of Moscatilin along with five clinically relevant drugs against two target proteins, viz., Anaphase-Promoting Complex subunit 10/Death of Cyclase 1 and Pyruvate Kinase Muscle isozyme M2 in silico. Out of five clinical ligands, four were plant-derived compounds, viz., Resveratrol, Paclitaxel, Shikonin, and Colchicine. The synthetic chemotherapeutic agent, Mitomycin-C, was used as a ligand to compare the mechanistic insights. The objective of the study was to determine the anticancer potency of Moscatilin in silico.

**Results:**

Moscatilin was found to have an advantage over other drugs of interest due to its structural simplicity and folding bridge connecting the bibenzyl structures. Moscatilin exhibited dual function by exclusively affecting the cancer cells, creating instabilities in biochemical and molecular cascades.

**Conclusions:**

The study demonstrates that Moscatilin is has a multi-antimetastatic function. Moscatilin interaction with APC10/DOC1 indicated that the drug is involved with post-replicative inhibition, and with PKM2 showed glycolytic pathway inhibition in cancer cells. Moscatilin can function as an effective cell cycle inhibitor.

**Graphical abstract:**

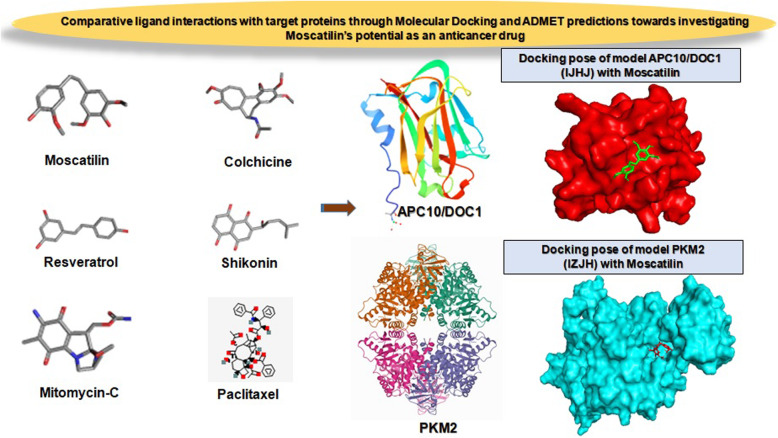

## Background

Disease complexity has always impelled researchers to focus on experimentation, directed towards drug discovery and its targeted delivery, notably in cancer. Synthetic drugs recommended for cancer display colossal side-effects. In today’s date, plant-based medicines serve decent effectiveness over synthetic equivalents or derivatives with minimal side-effects. Many phytochemicals, mostly in the form of anticancer compounds, have already been successfully established as anticancer drugs, viz., Camptothecin, Paclitaxel, Podophyllotoxin, Vinblastine, and Vincristine [1]. The present study is aimed at deriving the mechanistic insights of a potential anticancer agent, Moscatilin, a bibenzyl derivative, by paralleling it with few other critical plant-derived drugs such as Resveratrol, a stilbenoid; Paclitaxel, a tetracyclic diterpenoid; Colchicine, an alkaloid; and Shikonin, a naphthoquinone derivative. Mitomycin-C (a clinical chemotherapeutic drug) was also used to compare the cytotoxic effect of Moscatilin.

“Moscatilin” is a bibenzyl derivative, primarily present in the orchid genus, *Dendrobium*. Moscatilin functions as a potential anticancer agent and the research efforts on this compound have been increasing of late [2]. It was found to induce significant cytotoxicity in the FaDu (human hypopharyngeal squamosa carcinoma) cell lines and numerous other cancer cell lines through several mechanisms notably, apoptosis through deoxyribonucleic acid (DNA) damage, c-Jun N-terminal kinase (JNK)/stress-activated protein kinase (SAPK) activation and tubulin depolymerization [3–5]. Moscatilin has also been seen causing cell cycle blockade in the Gap 2/Mitosis (G2/M) phase along with mitotic catastrophe [6]. The compound was observed hindering metastasis and migration by inhibiting Akt and Twist signaling pathways in breast cancer cells [7]. Besides, Moscatilin repressed tumor angiogenesis and growth in human umbilical vein endothelial cells (HUVEC), halting endothelial nitric oxide synthase (eNOS), Extracellular signal-regulated protein kinases (ERK1/2), and Akt pathways [8]. Its significance lies in causing cytotoxicity in neoplasms at non-toxic concentrations [9]. The exact mechanisms of antitumor activity of Moscatilin are yet to be understood. Additionally, its potency needs to be compared too with biodrugs of clinical relevance.

Resveratrol (3,5,4′-Trihydroxystilbene) is essentially a polyphenol and a natural nutraceutical phytoalexin derived naturally from fruits such as grapes blueberries, cranberries, and also peanuts. It is a structural analog of Moscatilin and presence of both of them has been reported from an ornamental orchid, *Dendrobium ovatum* (a threatened species, endemic to the Western Ghats, India) and hybrids of *Dendrobium* [10]. Antioxidative, anticancer, and anti-angiogenic properties of Resveratrol have been widely reported [11, 12]. Colchicine disrupts tubular dynamics interrupting cell cycle progression. The capping of microtubules with Colchicine induces steric clashes, subsequently resulting in microtubular disassembly. Microtubular disassembly eventuates “c-mitosis” — an artificially induced mitosis, under the influence of Colchicine, where the nuclear division gets aborted, causing the doubling of chromosome number [13]. Paclitaxel (derived from the bark of plant, *Taxus brevifolia*) disturbs the dynamic activity of the microtubule, resulting in microtubular stabilization, obstructing the cell cycle at the mitotic (M)-phase [14]. Shikonin is derived from the roots of *Lithospermum erythrorhizon* and its anticancer, anti-inflammatory, and anti-obesity effects have been well-described [15]. This compound has been identified as a potent blocker of Pyruvate kinase Muscle isozyme M2 (PKM2) [16]. Shikonin also shows more affinity towards PKM2 than any of its isoforms, viz., pyruvate kinase-M1 (PKM1), Pyruvate kinase liver type (PKL), and Pyruvate kinase red blood cell type (PKR) [17]. Mitomycin-C is a vesicant, and it induces severe tissue injury when slips out of the vein. It is reported to cause damage to nucleic acids — both DNA and ribonucleic acid (RNA), resulting in the shrinkage of tumor cells [18, 19]. This clinical drug has numerous side effects, such as mouth sores, reduced appetite, fatigue, hair loss, diarrhoea, and bladder inflammation. Mitomycin-C has numerous functions starting from antibiotic properties to immunosuppression. It has been proven that Mitomycin-C can be applied extensively in vascularized composite allotransplantation (VCA), a clinical procedure by which allograft rejection can be prevented by suppressing the immunological responses in T-cells [20].

In the current study, we have performed the molecular docking studies of all the six compounds (Moscatilin, Resveratrol, Paclitaxel, Colchicine, Shikonin, and Mitomycin-C) with two relevant proteins, viz., Anaphase-Promoting Complex subunit 10/Death of Cyclase 1 (APC10/DOC1) and PKM2. APC is an ubiquitin ligase (E3) complex, operating at the metaphase-to-anaphase transition of the cell cycle [21]. It favors the polyubiquitination of the enzyme “securin”, an anaphase inhibitor, enabling “separase” to digest the “cohesins” that hold the sister chromatids together. Moreover, it also promotes the degradation of cyclin B, which is an activating subunit of cyclin-dependent kinase 1 (cdk1). It also performs the task of polyubiquitination at specific lysine residue on a target protein and is responsible for inducing somatic mutations in Cancer [22], whereas Pyruvate kinase converts Phosphoenolpyruvate (PEP) to Pyruvate during Glycolysis. The Pyruvate thus generated gets converted into Acetyl-CoA and joins the Citric acid cycle. Pyruvate kinase exists as isoforms such as PKM1, PKM2, PKR, and PKL and they are expressed differentially in various tissues and cell types. PKM2 exists as a highly active tetrameric form and a low active dimeric form. The rate-limiting step of Glycolysis is governed by this dimeric form of PKM2, which shifts the glucose metabolism from the typical respiratory cascade to lactate metabolism in tumor cells [23]. PKM2 also manages the metabolic processes associated with cancer cells, and its high expression has been reported in various cancer types [24]. Blocking of this protein in cancerous cells is significant, as it forms the rate-determining step towards the end of the glycolytic reaction, generating a considerable amount of energy. Hence, resisting this step would promote the cancer cells to become nutrition deficient, eventually leading to their death. PKM2 is involved in both glycolytic and non-glycolytic pathways, apart from playing a vital role in tumor malignancy. Therefore, it is regarded as one of the remarkable therapeutic targets of the cancer disease.

Overall, the present study has two major goals; firstly, to ascertain the efficacy of Moscatilin on two target proteins APC 10/DOC1 (PDB ID: 1JHJ) and PKM2 (PDB ID: 1ZJH) and secondly, to compare the potential of Moscatilin with structurally related ligand Resveratrol and clinically used ligands such as Paclitaxel, Colchicine, Shikonin, and Mitomycin-C. The study hypothesized that blocking the catalytic sites of the above two proteins by Moscatilin might disclose novel avenues proving its pertinence in cancer therapy. Moreover, this research investigation also includes the characterization and comparison of adsorption, distribution, metabolism, excretion, and toxicity (ADMET) profiles of Moscatilin and the other five ligands of interest.

## Methods

### Molecular docking studies

During any drug research, analyses of the binding affinity and the interactions involved are essential. These interactions generally involved ionic and hydrogen bonds along with Van der Waals and hydrophobic interactions. Overall, the interaction aspect constituted a significant part of molecular recognition. Docking studies were always carried out to identify the best-fit orientation of a protein and a ligand, estimating the stability of their association. In that context, the present study illustrated the binding affinity between a phytotherapeutic Moscatilin and two target proteins that are mainly found to be upregulated during cancer. The current study was intended towards testing the probable inhibitory character of Moscatilin against the target proteins to identify its multi-target nature in comparison with a few other clinical anticancer drugs.

### Preparation of ligands and target proteins for docking studies

Molecular docking was performed using the web servers, “PatchDock” (https://bioinfo3d.cs.tau.ac.il/PatchDock/) and “FireDock” (https://bioinfo3d.cs.tau.ac.il/FireDock/). Through PatchDock and FireDock, a two-tier approach was used to predict the ligand-target protein-interacting complexes to get a clear idea regarding the mechanism of action. The first step of docking was performed by retrieving the structures of both the target proteins from the Protein Data Bank (http://www.rcsb.org/). Then, the 3D structures of ligands were retrieved from the PubChem database (https://pubchem.ncbi.nlm.nih.gov/). The ligand structures in SDF formats were converted into PDB formats, and they were optimized. The “Clustering RMSD” was fixed to 4.0 Å for docking calculations. The ligands were prepared through energy minimization along with the addition of charges (for correcting ionization) and polar hydrogens. Structure optimization was done by assigning bond angles, bond orders, and topology. The proteins were prepared through the removal of heteroatoms (water molecules), irrelevant ions, and ligands. This was followed by uploading the PDB files of both protein and ligand to the “PatchDock” server. The relative efficacy of the compounds was projected by comparing the global energy derived from the analyses.

### Visualization of binding pockets

The results obtained from PatchDock were further refined by FireDock, uploaded to Protein-Ligand Interaction Profiler (PLIP), “Run Analysis” was clicked, and the protein-ligand docked structure was visualized in a three-dimensional space. PLIP is an automated tool meant for visualization and high-throughput analysis of relevant non-covalent interactions in 3D structures. On submission of the protein and ligand combination, PLIP delivers a set of marked interactions between the ligand and the protein, resulting in the stabilization of the system. Besides visualization by PLIP, the binding pockets of the ligand were again re-verified using a free academic version of PyMOL (https://pymol.org/2/).

### Interpreting the binding parameters

The interpretation was made based on the binding energies and the Van der Waals forces (VdW), both attractive and repulsive. Based on the scores obtained, the best solutions declared by the FireDock was again visualized using PyMOL to generate the dot surface and to study the ligand-protein interaction poses. The results obtained through docking were represented as e-negative values. Higher negative e-values indicate high ligand-protein binding affinity that represents higher efficiency of the phytochemicals. While looking for the ligand-protein interactions, the amino acid residues were analyzed to detect and interpret both the hydrophobic and hydrogen bond interactions.

### Assessment of drug-likeness and in silico ADMET prediction

Drug-likeness and the ADMET profiles were analyzed using admet structure-activity relationship (admetSAR) 2.0 tool/database (http://lmmd.ecust.edu.cn/admetsar2/) [25] and an online version of SwissADME web tool (http://www.swissadme.ch) [26]. For this analysis, the Simplified Molecular Input Line Entry System (SMILES) formats of all the ligands were obtained from PubChem database. Lipinski’s rule of 5 was applied towards the drug-likeness of all the ligands, to check if all the properties fall within the accepted range. Lipophilicity levels were analyzed based upon the atom-based logarithm of the partition coefficient (ALogP). The absorption of compounds (ligands) was analyzed by looking into the values associated with immortalized human colorectal adenocarcinoma cell line (Caco-2), permeability (P)-glycoprotein inhibitor/substrate, and human intestinal absorption (HIA). Blood-brain barrier (BBB) was checked towards the distribution of drugs. Drug metabolism was estimated based upon the Cytochrome P450 (CYP) models (CYP1A2, CYP2C19, CYP2C9, CYP2D6, and CYP3A4) for substrate or inhibition. Apart from these, drug toxicity was also analyzed, mainly considering human ether-a-go-go-related gene (hERG) inhibition, AMES toxicity, and hepatotoxicity. Comprehensively, all the significant ADMET parameters of the compound Moscatilin was estimated and checked towards compliance with their standard ranges for its identification as a suitable drug candidate. Additionally, to estimate the potential of Moscatilin, they were also compared with the critical parameters associated with other clinical drugs of the study.

## Results

The study focused on predicting the affinity of six ligands, viz., Moscatilin, Resveratrol, Paclitaxel, Colchicine, Shikonin, and Mitomycin-C, towards two target proteins, APC10/DOC1 and PKM2. The lesser the value of atomic contact energy (ACE), the more significant and useful would be the binding energy. The approximate interface area of the complex and ACE that PatchDock for the ligand-protein complex generated was further refined using FireDock.

### Binding of ligands and proteins

**Fig. 1:**
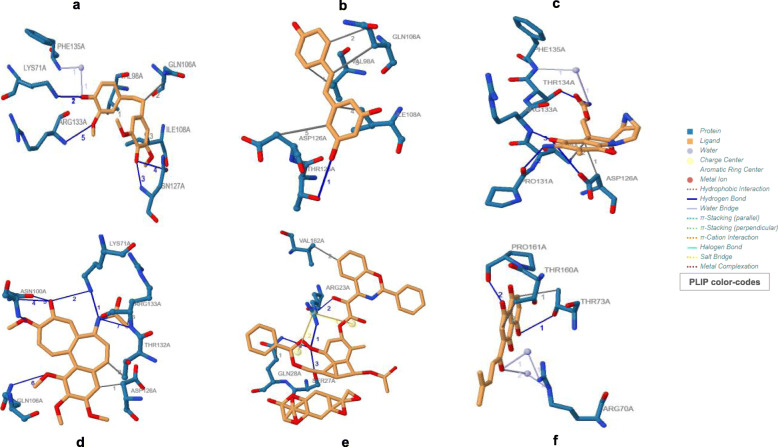
Ligands docked with target protein APC10/DOC1 (IJHJ), generated using PLIP. The ligands used were **a** Moscatilin, **b** Resveratrol, **c** Mitomycin-C, **d** Colchicine, **e** Paclitaxel, and **f** Shikonin

**Fig. 2:**
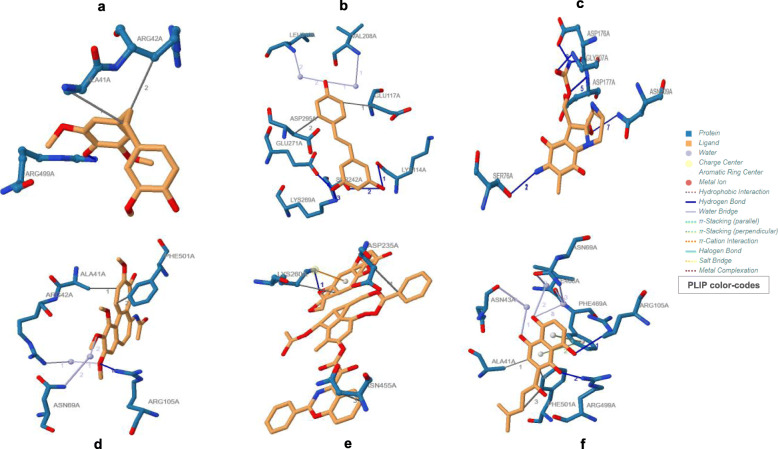
Ligands docked with target protein PKM2 (1ZJH), generated using PLIP. The ligands used were **a** Moscatilin, **b** Resveratrol, **c** Mitomycin-C, **d** Colchicine, **e** Paclitaxel, and **f** Shikonin

**Fig. 3:**
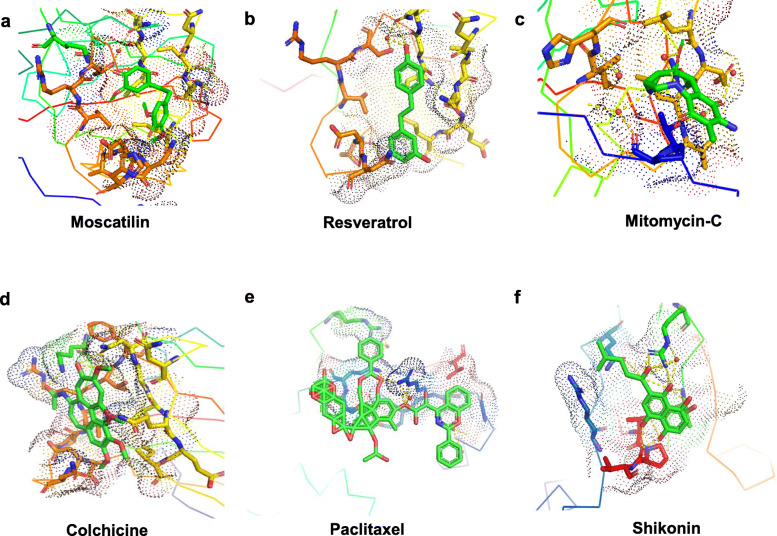
Interaction of ligands with target protein APC10/DOC1 (IJHJ). Docked positions of ligands (color — light green), generated by PyMOL. The ligands used were **a** Moscatilin, **b** Resveratrol, **c** Mitomycin-C, **d** Colchicine, **e** Paclitaxel, and **f** Shikonin

**Fig. 4:**
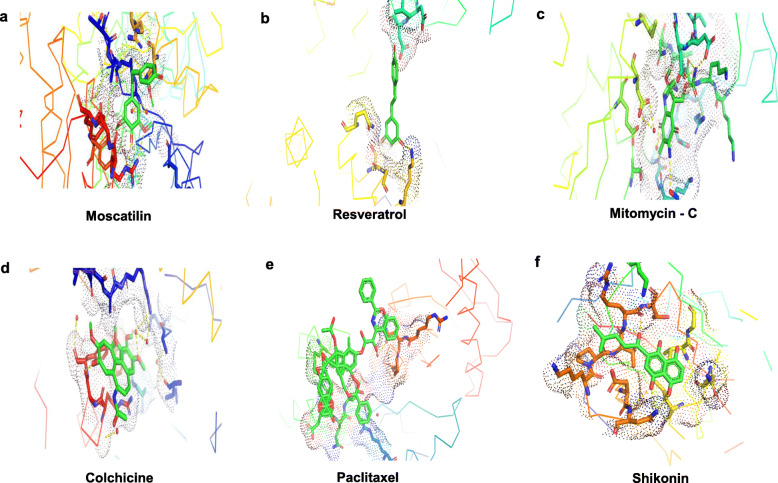
Interaction of ligands with target protein PKM2 (1ZJH). Docked positions of ligands (color — light green), generated by PyMOL. The ligands used were **a** Moscatilin, **b** Resveratrol, **c** Mitomycin-C, **d** Colchicine, **e** Paclitaxel, and **f** Shikonin

It was found that both Shikonin and Mitomycin-C displayed the least ACE towards APC10/DOC1 (Table 1). The value of ACE for Moscatilin was just below Shikonin and Mitomycin-C, exhibiting better binding affinity than the other three studied ligands, viz., Resveratrol, Colchicine, and Paclitaxel. But Moscatilin, in terms of its ACE, displayed only a moderate interaction with PKM2, when compared to compounds, viz., Paclitaxel, Shikonin, Colchicine, and Resveratrol. Out of all the ligands analyzed, Mitomycin-C displayed the least binding affinity towards PKM2 and Paclitaxel exhibited the highest affinity. However, it is the global energy that demonstrates the overall efficiency of ligand affinity towards a target protein. The lesser global energy, the more considerable is the interaction of the ligand. The global energy values towards both APC10/DOC1 and PKM2 indicated that Moscatilin was equally efficient as the clinical drug Mitomycin-C, because it was able to create perturbations on the contact surfaces of the protein. Resveratrol and Moscatilin were more efficient in interacting with PKM2 than Mitomycin-C, whereas Mitomycin-C and Moscatilin were more interactive towards APC10/DOC1 in comparison with Resveratrol. Based upon the global energy values, the interaction of Moscatilin towards APC10/DOC1 was on par with Shikonin and Mitomycin-C (Table 1). Automated prediction of protein-small molecule interactions has always posed challenges in the field of structural biology. Many docking algorithms have been developed to resolve these challenges, but they are computationally too heavy, demanding extensive experimental validation. PatchDock is a simple geometry-based molecular docking algorithm that comes up with near-native solutions and yielded molecular shape complementarity and steric clashes [27]. FireDock optimized binding energy through the refinement of ligand structure orientation, and this binding energy is expressed through attractive and repulsive Van der Waals (VdW) force [28]. The same for all the studied ligands corresponding to both the target proteins have been shown in Table 1. The global energy comparisons indicated that Moscatilin could inhibit the glycolytic pathway specific to cancer cells by interacting with PKM2. It also blocked the active site of APC10/DOC1 much more strongly when compared to PKM2 and seemed to preclude the function of APC in cancer cells. The active site perturbations of APC are probably the chief cause behind the post-replicative (G2/M phase) inhibition displayed by Moscatilin. The global energy values indicated that Resveratrol was less potent than Paclitaxel, Colchicine, and Shikonin towards PKM2. However, Resveratrol was found to be more effective towards PKM2 as compared to Moscatilin (Table 1). On a comparative analysis of minimum global energy, we find that the highest binding affinity supported Mitomycin-C, closely followed by Shikonin and Moscatilin towards APC10/DOC1. Paclitaxel displayed the most negligible binding affinity towards APC10/DOC1. The most frequently interacting (hydrophobic interactions) amino acid residue of APC10/DOC1 with Moscatilin was Valine (Fig. 1), and for PKM2, it was Arginine (Fig. 2). For Resveratrol, it was Isoleucine and Aspartic acid, respectively (Table 2). Mitomycin-C displayed hydrophobic interactions with residue Aspartic acid for APC10/DOC1 and had zero hydrophobic interaction with PKM2. The most frequently interacting (hydrophobic interactions) amino acid residue of APC10/DOC1 with Shikonin was Threonine. The most frequently interacting (hydrogen bond interactions) amino acid residue of APC10/DOC1 with Moscatilin was Asparagine, and for PKM2, it was Arginine (Table 3). For Resveratrol, it was Threonine, and for APC10/DOC1 and for PKM2, it was Lysine. Mitomycin-C displayed hydrogen bond formations with Aspartic acid residues of APC10/DOC1, whereas Asparagine residue of PKM2 formed hydrogen bonds with Mitomycin-C. Docked positions of all the six ligands of the study while interacting with both the target proteins (APC10/DOC1 and PKM2) were shown in Figs. 3 and 4. Regarding the hydrogen bond formation, both Moscatilin and Shikonin had the same residue interaction with PKM2, which is Arginine (Table 3). “Oxygen” of hydroxide moiety constantly interacted with Arginine residues, which would be the cause of a higher inhibitory effect of Shikonin towards PKM2. Surface interactions were more prominent for Paclitaxel, Colchicine, and Shikonin owing to their structural complexity. This structural intricacy led to supplementary resilient ligand interfaces with the target protein PKM2 (Table 4). The global energy minima related to PKM2 showed that Paclitaxel forms the most stable complex, followed by Colchicine, Shikonin, and Resveratrol. The high affinity of Paclitaxel towards PKM2 (1ZJH) could be because of the occurrence of π-cation interactions (Table 4).

**Table 1:** Protein-ligand interactions

Ligand	Target protein (PDB ID)	Global energy (Kcal/mol)	Attractive VdW (Kcal/mol)	Repulsive VdW (Kcal/mol)	Atomic contact energy (ACE) (Kcal/mol)
**Moscatilin**	**APC10/DOC1 (IJHJ)**	− 28.84	− 14.50	2.45	− 6.74
**Resveratrol**		− 22.62	− 10.65	0.62	− 6.14
**Mitomycin-C**		− 31.80	− 15.01	2.82	− 8.43
**Paclitaxel**		− 19.20	− 13.77	4.77	− 6.34
**Colchicine**		− 22.72	− 16.24	11.73	− 6.64
**Shikonin**		− 29.60	− 14.06	3.04	− 8.97
**Moscatilin**	**PKM2 (IZJH)**	− 28.91	− 14.46	5.23	− 8.23
**Resveratrol**		− 34.01	− 15.96	6.77	− 11.09
**Mitomycin-C**		− 26.37	− 14.99	3.71	− 4.85
**Paclitaxel**		− 59.86	− 26.79	11.38	− 18.73
**Colchicine**		− 36.51	− 16.23	6.07	− 11.10
**Shikonin**		− 36.31	− 13.91	2.77	− 11.44

**Table 2:** Stability assessments concerning hydrophobic interactions

Ligand	Target protein (PDB ID)	Residue	Amino acid	Distance (Å)	Ligand atom	Protein atom
**Moscatilin**	**APC10/DOC1 (IJHJ)**	98A	Valine	2.69	1462	764
**Resveratrol**		108A	Isoleucine	2.90	1458	845
**Mitomycin-C**		126A	Aspartic acid	3.24	1466	991
**Paclitaxel**		28A	Glutamine	2.85	1473	196
**Colchicine**		126A	Aspartic acid	2.73	1460	991
**Shikonin**		73A	Threonine	3.34	1457	564
**Moscatilin**	**PKM2 (IZJH)**	42A	Arginine	3.52	4010	147
**Resveratrol**		117A	Glutamine	3.63	4007	719
**Mitomycin-C**		No hydrophobic interactions				
**Paclitaxel**		455A	Asparagine	3.26	4050	3289
**Colchicine**		501A	Phenylalanine	2.66	4019	3657
**Shikonin**		41A	Alanine	2.87	4009	142

**Table 3:** Stability assessments with respect to hydrogen bonds.

Ligand	Target protein (PDB ID)	Residue	Amino acid	Distance H-A	Distance D-A	Protein donor	Side chain
**Moscatilin**	**APC10/DOC1 (IJHJ)**	127A	Asparagine	1.45	2.43	√	×
**Resveratrol**		125A	Threonine	3.05	4.01	√	√
**Mitomycin-C**		126A	Aspartic acid	3.22	4.00	√	√
**Paclitaxel**		28A	Glutamine	2.81	3.75	√	√
**Colchicine**		133A	Arginine	3.19	3.95	√	×
**Shikonin**		161A	Proline	1.66	2.29	×	×
**Moscatilin**	**PKM2 (IZJH)**	499A	Arginine	1.27	2.12	√	√
**Resveratrol**		269A	Lysine	1.15	2.14	√	√
**Mitomycin-C**		209A	Asparagine	2.10	3.08	√	√
**Paclitaxel**		260A	Lysine	2.89	3.26	√	√
**Colchicine**		105A	Arginine	3.10	4.00	√	√
**Shikonin**		499A	Arginine	2.07	2.70	√	√

**Table 4:** Additional resilient ligand-PKM2 interactions that differed from Moscatilin

Ligand	Target protein (PDB ID)	Mode of interaction	Residue	Amino acid	Group involved	Interacting ligand atom positions
**Colchicine**	**PKM2 (IZJH)**	Water bridge	42A	Arginine	Protein donor involved	4017
			69A	Asparagine		4016
**Paclitaxel**		π-cation interaction	260A	Lysine	Aromatic	4006, 4007, 4034, 4035, 4041, 4043
**Shikonin**		Water bridge	69A	Asparagine	Protein donor involved	3907
		π-stacking	469A	Phenylalanine	T-type	4005,4006,4007, 4008, 4009, 4010, 4011, 4012, 4013, 4014

### Evaluation of ADMET profiles of the ligands

**Fig. 5:**
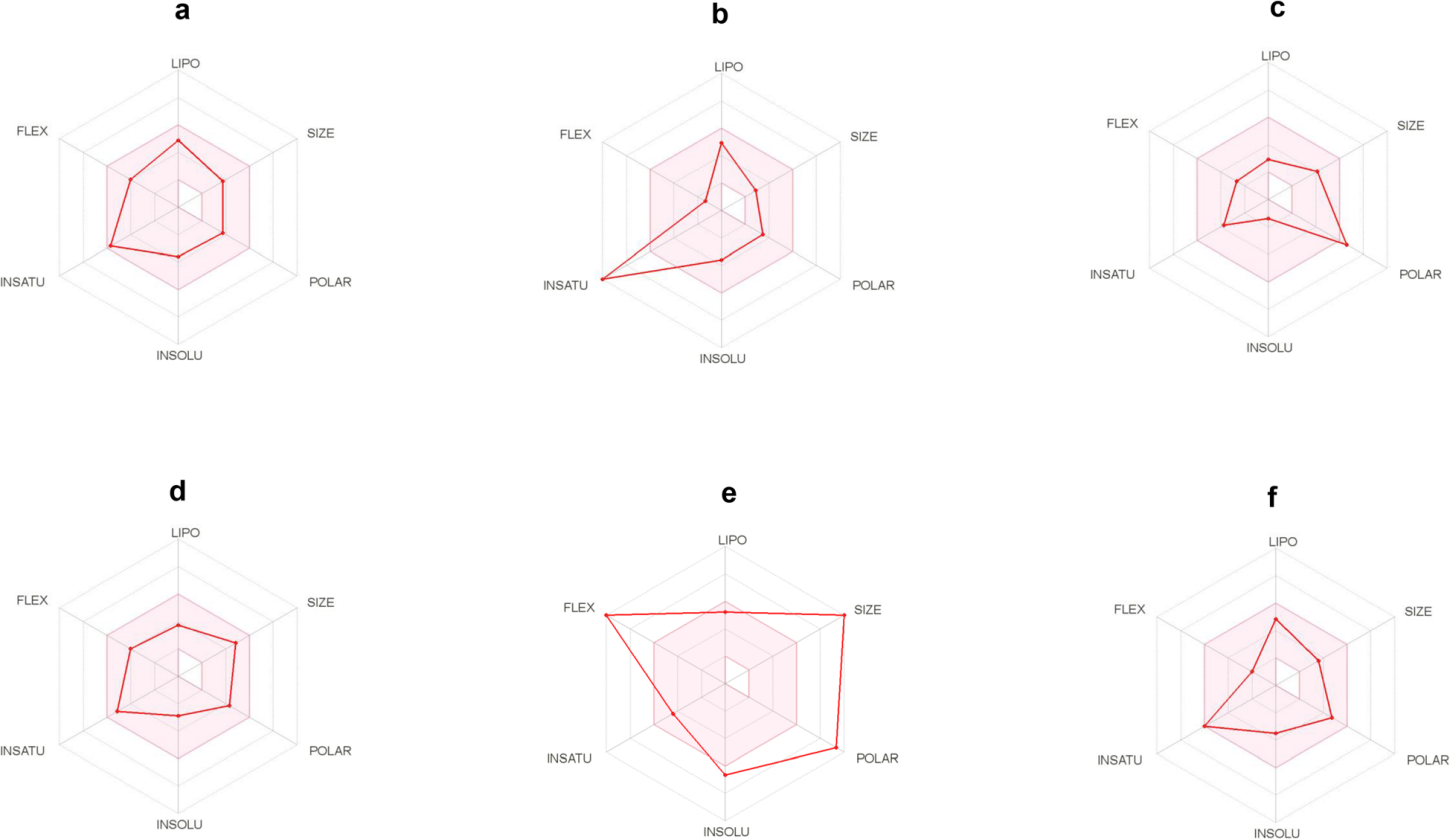
The bioavailability radar of the ligands, evaluated through SwissADME web tool. The ligands used were **a** Moscatilin, **b** Resveratrol, **c** Mitomycin-C, **d** Colchicine, **e** Paclitaxel, and **f** Shikonin. The colored zone specifies the relevant physicochemical space for oval bioavailability

Through the admetSAR and SwissADME analysis it was observed that Moscatilin follows Lipinski’s rule of five towards drug-likeness with molecular weight 304.34 (less than 500 g/mol) with two H-bond donor (not more than 5), five H-bond acceptor (not more than 10), AlogP value of 2.91 (not more than 5), 6 rotatable bonds (not more than 10), Topological Polar Surface Area (TPSA) of 68.15 Å^2^ (< 140 Å^2^), and molar refractivity of 84.22 (40–130). The low logP value of Moscatilin indicated good absorption and permeation with higher hydrophilicity. It was also found to be non-carcinogenic, non-AMES toxic, BBB positive, HIA positive, Caco-2 permeable along with negative hERG inhibition, negative for aromatase binding, micronuclear, and biodegradation (Table 5). HIA value of Moscatilin was found to be the second-highest among all the ligands with better BBB penetration. CYP2D6 and CYP3A4 are two main Cytochrome P450 enzymes that play significant roles during drug metabolism in the liver. The analysis identified Moscatilin as a CYP2D6 substrate/non-inhibitor and CYP3A4 non-substrate/non-inhibitor, indicating that the drug may be metabolized in the liver. Moscatilin was also identified as a P-glycoprotein non-substrate/non-inhibitor; therefore, it may not be easily transported in the body. The compound was identified with few toxicities such as acute oral, crustacean aquatic, fish aquatic, honey bee, hepatotoxicity, etc., but the values were found to be mostly lower than a few of the clinical drugs. SwissADME analysis indicated that Moscatilin followed all the drug-like filters, viz., Ghose, Veber, Egan, and Muegge that defined drug-likeness constraints through different parameters (Table 6). Bioavailability score for Moscatilin was observed as 0.55, which implied that it had 55% probability of rat bioavailability (higher than 10%). No alert was visualized for PAINS and Brenk, indicating the specificity of the compound. Moscatilin also exhibited leadlikeness and a lower value of synthetic accessibility in comparison with other studied ligands. The bioavailability radar of all the ligands with parameters such as size, lipophilicity, polarity, insolubility, insaturation, and flexibility are shown in Fig. 5.

**Table 5:** Selected in silico ADMET properties of all the ligands, including the probability

ADMET predicted profile — classifications	Ligands											
	Moscatilin		Resveratrol		Mitomycin-C		Colchicine		Paclitaxel		Shikonin	
	Value	Probability	Value	Probability	Value	Probability	Value	Probability	Value	Probability	Value	Probability
Ames mutagenesis	-	0.5700	-	0.8200	+	0.9100	-	0.9100	-	0.5383	+	0.5500
Acute Oral Toxicity (c)	III	0.7122	III	0.6825	I	0.7789	III	0.6116	III	0.5918	III	0.7812
Androgen receptor binding	+	0.5534	+	0.7659	+	0.8696	+	0.8697	+	0.8337	+	0.5547
Aromatase binding	-	0.5737	+	0.9242	-	0.5145	-	0.6515	+	0.6028	+	0.6380
Avian toxicity	-		-		-		-		-		-	
Blood Brain Barrier	+	0.8843	-	0.6616	-	0.9649	+	0.9821	-	0.9930	-	0.4134
BCRP inhibitor	-		-		-		-		-		-	
Biodegradation	-	0.8250	-	0.8750	-	0.8750	-	0.8500	-	0.8250	-	0.8750
BSEP inhibitor	-	0.6562	-	0.6594	-	0.7670	+	0.9192	+	0.9715	-	0.8073
Caco-2	+	0.7795	+	0.8398	-	0.6402	+	0.7766	-	0.9373	-	0.6593
Carcinogenicity (binary)	-	0.7714	-	0.5301	-	0.7316	-	0.8571	-	0.9286	-	0.8143
Carcinogenicity (ternary)	Non-required	0.6638	Non-required	0.5753	Danger	0.7522	Non-required	0.6626	Non-required	0.4813	Non-required	0.6524
Crustacea aquatic toxicity	+	0.5951	+	0.5600	-	0.7100	-	0.6400	+	0.5700	-	0.7700
CYP1A2 inhibition	+	0.6654	+	0.9106	-	0.5813	-	0.9045	-	0.9045	+	0.8668
CYP2C19 inhibition	+	0.7320	+	0.8052	-	0.6115	-	0.9025	-	0.9025	+	0.7648
CYP2C9 inhibition	-	0.6580	+	0.7068	-	0.7642	-	0.9071	-	0.9071	+	0.8714
CYP2C9 substrate	-	0.6120	-	0.5955	-	1.0000	-	1.0000	-	1.0000	-	0.6023
CYP2D6 inhibition	-	0.8154	-	0.9226	-	0.7464	-	0.9231	-	0.9231	+	0.6042
CYP2D6 substrate	+	0.5079	-	0.6927	-	0.8496	-	0.8323	-	0.8698	-	0.8340
CYP3A4 inhibition	-	0.8416	+	0.7539	-	0.8308	-	0.8310	-	0.8309	-	0.6708
CYP3A4 substrate	-	0.5851	-	0.7342	+	0.5998	+	0.7604	+	0.7980	-	0.5793
CYP inhibitory promiscuity	+	0.6541	+	0.8559	+	0.5204	-	0.7959	-	0.8937	+	0.7179
Eye corrosion	-	0.9598	-	0.9581	-	0.9886	-	0.9886	-	0.9872	-	0.9906
Eye irritation	+	0.8681	+	0.9960	-	0.9694	-	0.9171	-	0.9100	+	0.5625
Estrogen receptor binding	+	0.7617	+	0.9144	+	0.8905	+	0.8906	+	0.8148	+	0.5593
Fish aquatic toxicity	+	0.9115	+	0.9588	+	0.8586	+	0.7913	+	0.9852	+	0.9940
Glucocorticoid receptor binding	+	0.7529	+	0.7722	+	0.7165	+	0.8433	+	0.7632	+	0.8022
Honey bee toxicity	+	0.6820	+	0.6729	+	0.7354	+	0.5285	+	0.5672	+	0.8103
Hepatotoxicity	+	0.6250	+	0.6750	-	0.8000	+	0.8500	+	0.9500	+	0.8250
Human either-a-go-go inhibition	-	0.5785	-	0.8361	-	0.4008	-	0.4207	+	0.7442	-	0.7431
Human Intestinal Absorption	+	0.9864	+	0.9825	+	0.9381	+	0.9822	+	0.9676	+	0.9927
Human oral bioavailability	-	0.6429	-	0.6857	+	0.5857	+	0.6857	-	0.9143	-	0.6143
MATE1 inhibitor	-	0.9600	-	0.9800	-	0.8000	-	0.9800	-	0.8700	-	0.8400
Micronuclear	-	0.6741	-	0.5900	+	0.9500	+	0.6300	+	0.7300	-	0.5141
OATP1B1 inhibitor	+	0.8850	+	0.9414	+	0.9470	+	0.9308	-	0.7738	+	0.9391
OATP1B3 inhibitor	+	0.8824	+	0.9479	+	0.9355	+	0.9589	+	0.9479	+	0.9560
OATP2B1 inhibitor	-	0.8483	-	0.7145	-	0.8586	-	1.0000	-	1.0000	-	0.7124
OCT1 inhibitor	+		-		-		-		-		-	
OCT2 inhibitor	-	0.9250	-	0.9088	-	0.9250	-	0.9322	-	0.9500	-	0.9000
P-glycoprotein inhibitor	-	0.8745	-	0.9537	-	0.9166	-	0.8485	+	0.7874	-	0.9107
P-glycoprotein substrate	-	0.7666	-	0.9899	+	0.9298	+	0.9713	+	0.9535	-	0.9172
PPAR gamma	+	0.5336	+	0.9289	+	0.8707	+	0.6818	+	0.8011	+	0.8189
Plasma protein binding	1.033		0.677		0.521		0.485		0.999		0.767	
Subcellular localization	Mitochondria		Mitochondria		Mitochondria		Nucleus		Mitochondria		Mitochondria	
*Tetrahymena pyriformis*	0.753		0.403		1.099		0.162		1.075		0.51	
Thyroid receptor binding	+	0.8532	+	0.7160	+	0.5411	+	0.8461	+	0.7275	-	0.5853
UGT catalyzed	+	0.8000	+	0.7000	-	0.0000	-	0.0000	+	0.6000	+	0.6000
Water solubility	− 3.747		− 2.778		− 2.682		− 2.561		− 3.873		− 3.832	

*BCRP* Breast Cancer Resistant Protein, *BSEP* Bile Salt Export Pump, *Caco-2* Cancer coli-2, *CYP* Cytochrome P450, *MATE-1* Multidrug and Toxin Extrusion, *OATP1B1* Organic Anion Transporter Protein B1, *OATP1B3* Organic Anion Transporting Polypeptide 1B3, *OATP2B1* Organic Anion Transporting Polypeptide 2B1, *OCT* Organic Cation Transporter, *P-glycoprotein* Permeability glycoprotein, *PPAR* Peroxisome Proliferator-Activated Receptor, *UGT* Uridine diphosphate glucuronosyltransferase

**Table 6:** Physicochemical properties and pharmacokinetics prediction of all the six ligands of the study by SwissADME

	Ligands					
	Moscatilin	Resveratrol	Mitomycin-C	Colchicine	Paclitaxel	Shikonin
**Physicochemical properties**						
Number of H-bond donors	2	3	4	6	15	3
Number of H-bond acceptors	5	3	3	1	4	3
Number of rotatable bonds	6	2	6	6	14	5
Molar refractivity	84.22	67.88	86.95	109.36	218.96	77.82
TPSA	68.15 Å^2^	60.69 Å^2^	146.89 Å^2^	83.09 Å^2^	221.29 Å^2^	94.83 Å^2^
**Pharmacokinetics**						
GI absorption	High	High	Low	High	Low	High
Log K_P_ (skin permeation) in cm/s	− 6.03	− 5.47	− 8.62	− 8.01	− 8.91	− 5.96
**Druglikeness**						
Lipinski	Yes	Yes	Yes	Yes	No	Yes
Ghose	Yes	Yes	No	Yes	No	Yes
Veber	Yes	Yes	No	Yes	No	Yes
Egan	Yes	Yes	No	Yes	No	Yes
Muegge	Yes	Yes	Yes	Yes	No	Yes
Bioavailability score	0.55	0.55	0.55	0.55	0.17	0.55
**Medicinal Chemistry**						
PAINS	0 alert	0 alert	1 alert	0 alert	0 alert	2 alerts
Brenk	0 alert	1 alert	2 alerts	0 alert	2 alerts	2 alerts
Leadlikeliness	Yes	No	Yes	No	No	Yes
Synthetic accessibility	2.23	2.02	4.80	3.87	8.34	3.55

*H-bond* hydrogen bond, *TPSA* topological polar surface Area, *GI* gastrointestinal, *K*_*p*_ permeability coefficient, *PAINS* pan-assay interference compounds

## Discussion

The in silico data indicated that Moscatilin is more of a cell cycle influencer yet moderately perturbing the glycolysis pathway. In contrast, the function of Resveratrol is just the opposite. Resveratrol influenced the biochemical pathways of Cancer, which aligns with various studies proposed elsewhere [29]. The inhibitory function of Shikonin on PKM2 has been well established through various wet-lab experiments, and the same is also confirmed through the in silico data obtained in the present study [30]. Moscatilin exhibited dual function by affecting the cancer cells exclusively, creating instabilities both in biochemical (glycolytic) and molecular (anaphase separation of chromosomes) cascades. Moscatilin might be an ideal candidate to test its influence on PKM2, which is seen exclusively in cancer cells. The influence of specific proteins might be the cause of the specificity of Moscatilin towards cancer cells. This perceptive, however, must be validated using wet-lab experiments. Apart from the Arginine interaction, the binding of the aromatic ring in the hydrophobic cavity disrupted the placement of the ionic group. The aromatic group interacted with positively charged residues in a protein such as an Arginine or a Lysine [31]. This interaction was seen prevalent in the case of Moscatilin in the current study. Both Moscatilin and Resveratrol being polyphenols shared almost similar structural configurations. However, Moscatilin had a structural benefit over Resveratrol due to a flexible chemical bond bridge connecting the two benzyl moieties. The interacting amino acid residues also implicated that cell cycle instability is a prominent function of Moscatilin than biochemical interferences. The π-cation interactions profoundly influenced the structural orientation and molecular recognition, and it impacts the catalytic activity [32]. As it had a catalytic impact, it caused changes in protein physiology. π-cation interactions were subjugated by the electrostatic attraction between an electron-rich arene and electron-deficient cation [33]. Shikonin had both water bridge and π stacking interactions with PKM2. π stacking refers to attractive non-covalent interactions between aromatic rings [34]. Non-covalent forces are of substantial importance to ligand loading in drug-delivery methods [35]. In addition to the non-destructive linking of the delivery vehicle and lodger drug, they provide multiple advantages such as protecting the structure and function of the drug apart from assisting its discharge towards the precise target.

However, π stacking interactions are vulnerable to pH and other exterior cues [36]. In comparison, Colchicine displayed water bridge interactions alone (Table 4). Water influences the ligand-protein binding energetics and contributes to the desolvation of the protein upon binding [37]. The presence of the water bridge in the microenvironment sites is indicative of translational and rotational diffusion rates and thermodynamics of the interacting molecules. The water molecules provide additional hydrogen bonding, which improves the binding affinity of the interacting surfaces, furthermore increasing the accuracy of the docking scores. The presence of water also has a profound influence on the orientational entropy of the interacting surfaces [38]. Their scores drastically differ if the water molecules are connected to charged atoms, which aid to compute the global energy of the protein-ligand complex. Thus, the presence of a water bridge impacts ligand geometry in the hydrophobic cavities, thereby playing a crucial role in shortlisting unique efficacious drug candidates. Systematic analysis of crystal structures showed that other factors, such as competitive hydrogen bonding interactions disconnected to the π-cation interaction or π stacking, might also affect the geometry, which is the case with Moscatilin. Studies related to the prediction of efficacy or competence and the absence of toxicity in the drug candidates are essential in the early in vitro studies of drug development, ensuring a higher success rate. To rapidly identify these efficient plant-derived drugs and their precise disease targets, in silico techniques are often chosen. Computational methods, notably molecular docking, hasten drug target identification. Molecular docking is a required method in structure-based drug design that estimates the binding affinity between two molecules. This binding nature assists in describing the critical biochemical processes related [39].

The present research investigation has identified the potential of Moscatilin as a promising drug candidate after the comparison of all the ADMET properties of this bibenzyl compound with the clinical drugs of the study. The results included in this research is based on the in silico approaches. Molecular dynamics and simulation studies need to be performed for more vital information. The study also needs in vitro and in vivo animal studies for the confirmation of Moscatilin as a potent inhibitor of APC10/DOC1 and PKM2 towards cancer treatment.

## Conclusion

Among the ligands tested, Moscatilin holds promise as an efficient chemotherapeutic agent. For target protein APC10/DOC1 (1JHJ), Moscatilin works as efficiently as Mitomycin-C and Shikonin in terms of minimal global energy. Regarding the target protein, PKM2 (1ZJH), we can conclude that Moscatilin and Resveratrol correspondingly participate in the interaction in terms of minimal global energy. The structural simplicity of Moscatilin and Resveratrol, along with their aromaticity, offers high lipophilicity to these plant-derived polyphenols. The flexibility of the chemical bond in Moscatilin that connects the aromatic structures and its interacting residues might unlock many more prospects in targeted chemotherapy in the future. The ADMET study affirms that Moscatilin with an excellent pharmacokinetic profile holds the potential as a suitable anticancer drug candidate. Moscatilin could be safe for healthy cells, as it showed specific interactions with proteins that get explicitly expressed in cancerous conditions, as seen in PKM2. Moscatilin is a safe drug for normal cells and acts explicitly on cancer cells [40]. However, Moscatilin is marketed only by a few pharmaceutical companies in China, and the current cost is $649 for 10 mg. The price is expected to mount after the compound has been established as a potential clinical drug after the clinical phase trials. The chemical synthesis of this phytometabolite has been quite expensive due to the requirement of starting material, and the process is quite tedious. *Dendrobium* genus is the only source where it can be extracted from. The study emphasizes the need for tissue culture strategies for conserving the genus and also invent methods to upscale the content of Moscatilin in vitro.

## Data Availability

The datasets used and/or analyzed during the current study are available from the corresponding author on reasonable request.

## Abbreviations

ADMET: Absorption, distribution, metabolism, excretion, and toxicity
APC10: Anaphase-Promoting Complex subunit 10
DOC1: Death of Cyclase 1
FireDock: Fast interaction refinement in molecular docking
PDB: The Protein Data Bank
PKM2: Pyruvate Kinase Muscle isozyme M2
PLIP: Protein-Ligand Interaction Profiler
RCSB: Research Collaboratory for Structural Bioinformatics
RMSD: Root-mean-square deviation
